# Mechanical Stretch Control of Adipocyte AKT Signaling and the Role of FAK and ROCK Mechanosensors

**DOI:** 10.3390/bioengineering11121279

**Published:** 2024-12-16

**Authors:** Tasneem Bouzid, Eunju Kim, Brandon D. Riehl, Ruiguo Yang, Viswanathan Saraswathi, Jason K. Kim, Jung Yul Lim

**Affiliations:** 1 Department of Mechanical and Materials Engineering, University of Nebraska-Lincoln, Lincoln, NE 68588, USA; tasb2010@yahoo.com (T.B.); nesprin1747@gmail.com (E.K.); briehl4@unl.edu (B.D.R.); yangruig@msu.edu (R.Y.); 2 Nebraska Center for Integrated Biomolecular Communication, University of Nebraska-Lincoln, Lincoln, NE 68588, USA; 3 Department of Biomedical Engineering, Michigan State University, East Lansing, MI 48824, USA; 4 Institute for Quantitative Health Science and Engineering (IQ), Michigan State University, East Lansing, MI 48824, USA; 5 Department of Internal Medicine, University of Nebraska Medical Center and VA Nebraska-Western Iowa Health Care System, Omaha, NE 68105, USA; s.viswanathan@unmc.edu; 6 Program in Molecular Medicine and Division of Endocrinology, Metabolism and Diabetes, Department of Medicine, University of Massachusetts Chan Medical School, Worcester, MA 01605, USA; jason.kim@umassmed.edu; 7 Nebraska Center for the Prevention of Obesity Diseases, University of Nebraska-Lincoln, Lincoln, NE 68588, USA

**Keywords:** adipocyte, mechanical stretch loading, insulin signaling, AKT, GLUT4, FAK, ROCK

## Abstract

Adipose tissue in vivo is physiologically exposed to compound mechanical loading due to bodyweight bearing, posture, and motion. The capability of adipocytes to sense and respond to mechanical loading milieus to influence metabolic functions may provide a new insight into obesity and metabolic diseases such as type 2 diabetes (T2D). Here, we evidenced physiological mechanical loading control of adipocyte insulin signaling cascades. We exposed differentiated 3T3-L1 adipocytes to mechanical stretching and assessed key markers of insulin signaling, AKT activation, and GLUT4 translocation, required for glucose uptake. We showed that cyclic stretch loading at 5% strain and 1 Hz frequency increases AKT phosphorylation and GLUT4 translocation to the plasma membrane by approximately two-fold increases compared to unstretched controls for both markers as assessed by immunoblotting (*p* < 0.05). These results indicate that cyclic stretching activates insulin signaling and GLUT4 trafficking in adipocytes. In the mechanosensing mechanism study, focal adhesion kinase (FAK) inhibitor (FAK14) and RhoA kinase (ROCK) inhibitor (Y-27632) impaired actin cytoskeleton structural formation and significantly suppressed the stretch induction of AKT phosphorylation in adipocytes (*p* < 0.001). This suggests the regulatory role of focal adhesion and cytoskeletal mechanosensing in adipocyte insulin signaling under stretch loading. Our finding on the impact of mechanical stretch loading on key insulin signaling effectors in differentiated adipocytes and the mediatory role of focal adhesion and cytoskeleton mechanosensors is the first of its kind to our knowledge. This may suggest a therapeutic potential of mechanical loading cue in improving conditions of obesity and T2D. For instance, cyclic mechanical stretch loading of adipose tissue could be explored as a tool to improve insulin sensitivity in patients with obesity and T2D, and the mediatory mechanosensors such as FAK and ROCK may be targeted to further invigorate stretch-induced insulin signaling activation.

## 1. Introduction

Obesity has reached epidemic levels in recent decades, increasing the risk of diseases such as hypertension, fatty liver disease, cardiovascular disease, diabetes, stroke, dementia, and cancer. Specifically, it was estimated that 1 in 10 Americans had diabetes and over 1 in 3 had prediabetes in 2018 [1], with a constant increasing trend corresponding to the growing rate of obesity. Type 2 diabetes (T2D) comprising more than 90% of all diabetes is characterized by insulin resistance, hyperglycemia, and in severe cases, inadequate pancreatic insulin production [2]. In healthy cells, insulin binds to insulin receptor (IR) in the plasma membrane and triggers downstream cascades, including AKT (or protein kinase B, PKB). AKT activation via phosphorylation induces the translocation of glucose transporter protein type-4 (GLUT4)-containing vesicles to the plasma membrane as a required step for glucose uptake. The role of AKT in insulin signaling and glucose homeostasis has been well established. For example, AKT-deficient mice developed peripheral insulin resistance and non-suppressible glucose production [3]. Obesity is associated with a chronic state of adipose tissue inflammation that leads to changes in cytokine secretions by adipocytes and macrophages [4], thereby resulting in various metabolic dysfunctions, including malfunctions in insulin signaling. Excessive extracellular matrix (ECM) protein deposition and fibrosis in adipose tissue in obesity, associated with hypertrophic and hyperplastic adipocyte growth, exacerbate the inflammatory status of the adipose tissue, further contributing to insulin resistance [5].

In addition to static extracellular mechanophysical conditions from ECM deposition and fibrosis, adipose tissue is exposed to compound dynamic mechanical loading cues—tensile, compressive, and shear forces due to bodyweight, weight bearing, posture, and motion [6]. Research has highlighted the role of mechanosensing in adipocytic functions, particularly in the adipogenic commitment and differentiation of adipocytic precursor cells. For example, cyclic stretching upregulated osteogenesis at the expense of adipogenesis in mesenchymal stem cells (MSCs) [7]. In our prior study, cyclic stretch loading inhibition of MSC adipogenic commitment was mediated by extracellular signal-regulated kinase (ERK) [8]. While these results support the mechanosensing capability of adipocyte precursors, little is known with respect to the mechanotransduction of fully differentiated mature adipocytes. Unlike adipocytic precursor cells, differentiated adipocytes are especially characterized with capabilities of lipid storage and metabolic regulation. This study uniquely investigates how differentiated adipocytes sense and respond to external mechanical loading to alter metabolic actions such as insulin signaling cascades.

We report the impact of physiologically relevant mechanical stretch loading on key insulin signaling cascades in differentiated adipocytes and the potential mechanosensors involved. We evidenced that cyclic stretching induces the phosphorylation of crucial insulin signaling effector AKT, as well as GLUT4 translocation to the plasma membrane. We also showed that mechanical stretch regulation of AKT activation in adipocytes is mediated by the focal adhesion and cytoskeletal mechanosensors focal adhesion kinase (FAK) and RhoA kinase (ROCK). Our proof-of-concept demonstration may open a new horizon on how differentiated adipocytes respond to dynamic mechanical loading to regulate insulin metabolic functions.

## 2. Materials and Methods

### 2.1. Adipocyte Culture and Mechanical Stretch Loading

Preadipocytes (3T3-L1, ATCC, CL-173, Manassas, VA, USA) were cultured on type-I-collagen-coated 6-well stretch plates (Bioflex, Flexcell, Burlington, NC, USA) using growth medium (GM) consisting of high-glucose Dulbecco’s modified Eagle’s medium (Gibco, Waltham, MA, USA), 10% fetal bovine serum (Gibco, Waltham, MA, USA), and 1% penicillin–streptomycin (Gibco, Waltham, MA, USA), Adipogenic differentiation and stretch loading were conducted inside the incubator using our protocols [8]. After 2 days of culture, adipogenesis was induced for 3 days by replacing GM with adipogenic induction medium, i.e., GM containing 10 μg/mL insulin (I9278, Sigma, Louis, MO, USA), 10 μM dexamethasone (D4902, Sigma, Louis, MO, USA), and 0.5 mM 3-isobutyl-1-methylxanthine (I7018, Sigma, Louis, MO, USA). Adipogenic induction was followed by adipogenic maintenance by changing the medium into adipogenic maintenance medium containing 10 μg/mL insulin only. Medium was replaced every 2–3 days for 6–7 days until about 90% of cells displayed accumulated lipid droplets. Adipogenesis was determined by measuring lipid accumulation by oil red O staining and the protein levels of FAS and C/EBPα by immunoblotting (Supplementary Figure S1). Differentiated adipocytes were exposed to stretch loading using computer-controlled cell-stretching equipment (FX-5000, Flexcell, Burlington, NC, USA), which equiaxially stretches cell-seeded elastic membrane over a circular loading post [8]. Cyclic stretch loading was applied for 2 h at 5% strain and 1 Hz frequency regimens that are physiologically relevant for adipose tissue in vivo [6,9]. Control adipocytes were differentiated on the same stretch plate under the same culture conditions but not stretched.

### 2.2. Immunoblotting

After stretching, key insulin signaling protein expression and phosphorylation were assessed by Western blotting. Antibodies for AKT (MAB2055, R&D Systems, Minneapolis, TX, USA), phospho-AKT (p-AKT at Ser473) (AF887, R&D Systems, Minneapolis, TX, USA), GLUT4 (G4173, Sigma, Louis, MO, USA), and GAPDH (SC-32233, Santa Cruz, Dallas, TX, USA) were used. Immunoblot intensities after chemiluminescence were quantified using NIH ImageJ (1.53q version) and normalized to the GAPDH loading control.

### 2.3. Inhibitors

To evaluate the effect of inhibiting FAK and ROCK on stretch regulation of AKT, FAK inhibitor (FAK14, 10 μM, SC-203950, Santa Cruz, Dallas, TX, USA) and ROCK inhibitor (Y-27632, 10 μM, S104910MM, Selleck, Houston, TX, USA) were added to cells just prior to stretch loading. The concentrations were chosen in the range that has the efficacy in disrupting focal adhesion and cytoskeletal dynamics without inducing cytotoxic effects based on our previous studies [10,11].

### 2.4. Cell Surface Biotinylation

The cell surface biotinylation assay was chosen to specifically isolate and quantify plasma-membrane-localized proteins, from which GLUT4 expression at the plasma membrane was determined in comparison with total cellular GLUT4 levels. The experiment was performed according to the protocol by Choi et al. [12]. Immediately after stretch loading exposure, cell-surface-expressed proteins were bound with Sulfo-NHS-SS-Biotin (21335, Pierce Biotechnology, ROCKFORD, IL, USA) for 30 min in phosphate-buffered saline (PBS, 10010023, Gibco, Waltham, MA, USA). Cells were washed with PBS and biotin-labeled cells were lysed with radioimmunoprecipitation assay (RIPA) buffer (89900, Thermo Scientific, Waltham, MA, USA). Lysed proteins were incubated for 2 h with high-capacity avidin–agarose resin (29204, Thermo Scientific, Waltham, MA, USA). After washing with RIPA buffer, agarose-bound proteins were extracted by the sample buffer, and plasma-membrane-surface-expressed GLUT4 was determined by immunoblotting.

### 2.5. Immunofluorescence and Analysis

Immunofluorescence was used to visualize spatial distribution and subcellular localization of GLUT4 and cytoskeletal components under stretching conditions. Antibody concentrations and imaging parameters were optimized based on manufacturer’s guidelines and previous applications in similar cellular models [13]. Cells were fixed with 4% formaldehyde solution and permeabilized with 0.1% Triton X-100 solution. For actin and lipid imaging, cells were stained with rhodamine–phalloidin (1:100, R415, Invitrogen, Carlsbad, CA, USA), LipidSpot (1:1000, 70069, Biotium, Fremont, CA, USA), and DAPI (1:1000, sc-3598, Santa Cruz, Dallas, TX, USA). To detect GLUT4, an immunofluorescence analysis was performed using a polyclonal anti-GLUT4 antibody (1:100, G4173, Sigma, Louis, MO, USA). GLUT4 immunofluorescent intensity was assessed with a Zeiss LSM800 confocal microscope (20× objective) driven by a ZEN confocal image acquisition program under unchanged image acquisition settings for all the samples. In ImageJ, GLUT4 fluorescent intensity was assessed following a line drawn radially from inside the cell and across the cell membrane (Supplementary Figure S2). Using the Plot Profile tool, fluorescent intensity was plotted as a function of location (in μm) converted from pixels.

### 2.6. Statistics

Statistical analysis was performed using MATLAB (R2015b, MathWorks, Natick, MA, USA). Data are expressed as mean ± standard error of measurement (SEM) from at least three independent experiments. Group comparisons were conducted using Student’s *t*-tests for pairwise comparisons or one-way analysis of variance (ANOVA) followed by Fisher’s least significant difference (LSD) post hoc tests for multiple group comparisons. Statistical significance is indicated as follows: *: *p* < 0.05, **: *p* < 0.01, and ***: *p* < 0.001.

## 3. Results

### 3.1. Cyclic Stretch Loading Upregulates AKT Phosphorylation in Differentiated Adipocytes

**Figure 1:**
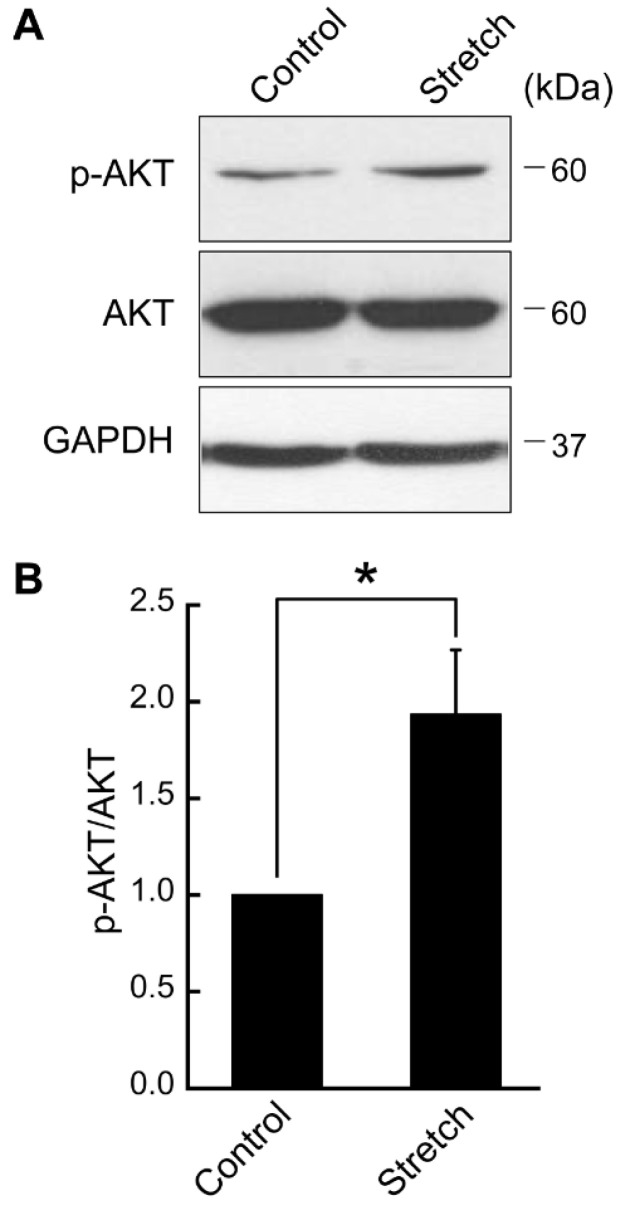
**Cyclic mechanical stretch loading induces AKT phosphorylation (p-AKT at Ser473) in adipocytes.** Cyclic stretching at 5% strain and 1 Hz frequency was applied to differentiated 3T3-L1 adipocytes for 2 h. (**A**) Immunoblots of AKT and p-AKT with GAPDH loading control. (**B**) After normalization with GAPDH, relative immunoblot band intensities of p-AKT/AKT were obtained. Mean ± SEM; *: *p* < 0.05 (n = 3) by Student *t*-test.

Here, we examined the impact of cyclic stretch loading on key insulin signaling effectors in mature adipocytes. First, differentiated adipocytes were cyclically stretched (5% strain, 1 Hz frequency) and AKT expression and phosphorylation were assessed (Figure 1). Cyclic stretch loading in these regimens triggered the phosphorylation of AKT (p-AKT at Ser473) in differentiated adipocytes, significantly increasing the relative level of p-AKT/AKT by approximately two-fold (Figure 1).

### 3.2. Cyclic Stretching Induces GLUT4 Translocation to the Plasma Membrane in Adipocytes

**Figure 2:**
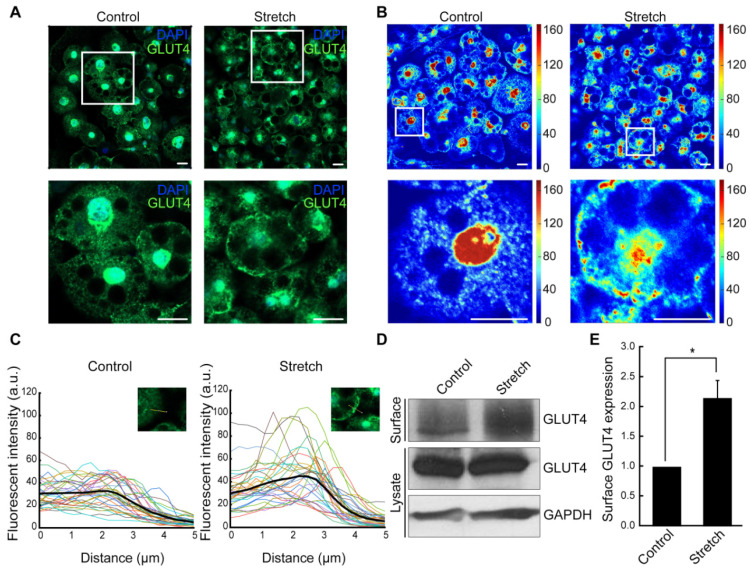
**Cyclic stretching of adipocytes induces GLUT4 localization in the plasma membrane.** (**A**) Adipocyte immunostaining with anti-GLUT4 antibody and DAPI. Scale bar: 20 µm. (**B**) Spatial intensity mapping of GLUT4, in which the intensity extracted from the immunofluorescence in (**A**) was shown by different colors (arbitrary unit). Scale bar: 20 µm. In (**A**,**B**), white boxes are magnified in the bottom rows. (**C**) The intensity of GLUT4 expression was traced across the plasma membrane (see Supplementary Figure S2 for detailed methods). The average GLUT4 intensity track is shown with a thick black line that is averaged from each measurements shown as different colored lines (n = 32). (**D**) Cell surface biotinylation assay was performed to conduct the immunoblotting of plasma-membrane-bound GLUT4. (**E**) The band intensity of membrane-surface-bound GLUT4 was normalized to the total GLUT4 expression. Mean ± SEM; *: *p* < 0.05 (n = 3) by Student *t*-test.

We then investigated whether cyclic stretch loading affects membrane translocation of GLUT4, a required step for insulin-mediated glucose uptake. In the unstretched control, GLUT4 was mainly located in the cytoplasm and the perinuclear region (Figure 2A). Stretched adipocytes displayed substantially increased GLUT4 localization in the plasma membrane, as shown by immunofluorescence (Figure 2A) and spatial intensity mapping (Figure 2B). In fluorescence intensity tracing curves (Figure 2C), while the GLUT4 intensity in unstretched adipocytes diminishes rather uniformly across the plasma membrane, stretched adipocytes show intensity peaks near the plasma membrane before sharply dropping. Biotinylation followed by immunoblotting further confirmed the GLUT4 plasma membrane localization (Figure 2D,E). Stretched adipocytes showed ca. two-fold increase in membrane surface expression of GLUT4 compared with the unstretched control, while GLUT4 expression in total cell lysates remained unchanged. These results indicate that mechanical stretch loading promotes GLUT4 translocation to the plasma membrane in differentiated adipocytes without affecting GLUT4 expression level.

The observed increase in GLUT4 translocation suggests that mechanical stretch may enhance insulin responsiveness in adipocytes potentially via increased GLUT4 trafficking to the plasma membrane and associated glucose uptake. This effect is particularly relevant to metabolic diseases such as obesity and T2D, where insulin resistance is characterized by impaired GLUT4 trafficking and reduced glucose uptake [14].

### 3.3. FAK and ROCK Regulate Actin Development and Mechanical Stretch Loading-Induced AKT Activation in Adipocytes

**Figure 3:**
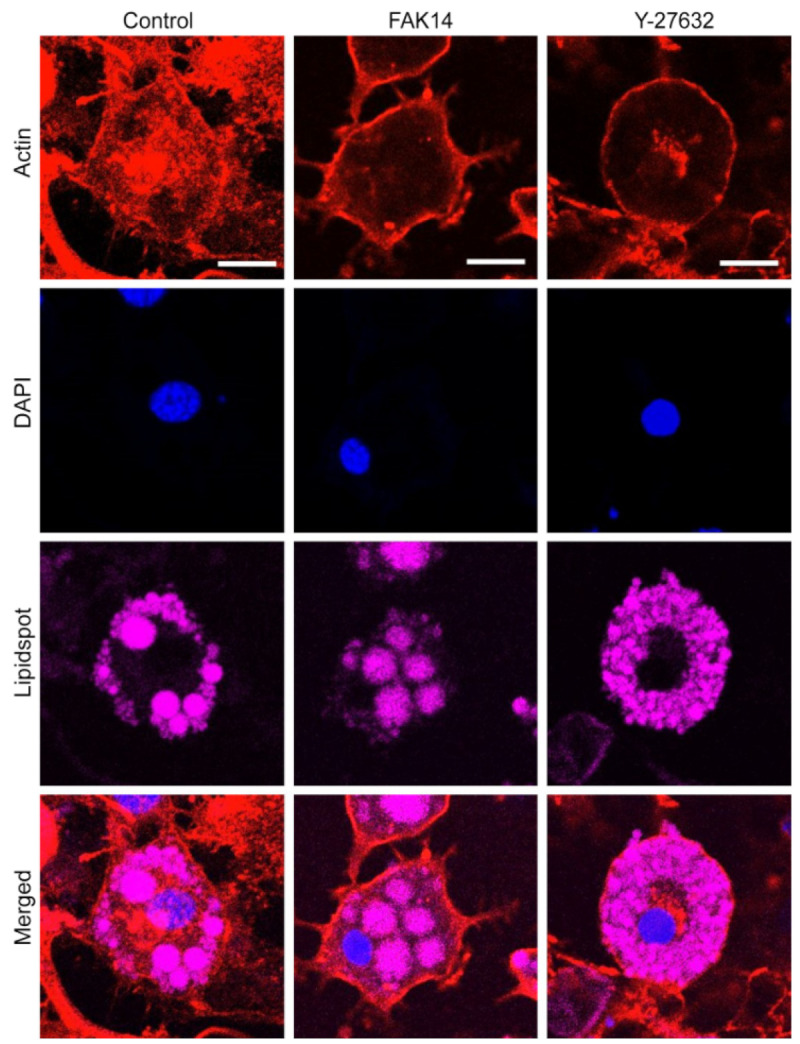
**FAK and ROCK inhibitors disrupt actin network formation in stretched adipocytes.** Adipocytes were stretched without or with FAK inhibitor (FAK14) and ROCK inhibitor (Y-27632). F-actin (red), DAPI (blue), and lipid (pink) staining. Scale bar: 20 µm.

As potential mechanosensing mechanisms of adipocyte response to mechanical stretch loading in AKT-associated insulin signaling, FAK focal adhesion and ROCK cytoskeletal signaling cascades were investigated. In immunofluorescent imaging, the FAK inhibitor (FAK14) and ROCK inhibitor (Y-27632) led to marked disruption in actin network formation in stretched adipocytes (Figure 3).

**Figure 4:**
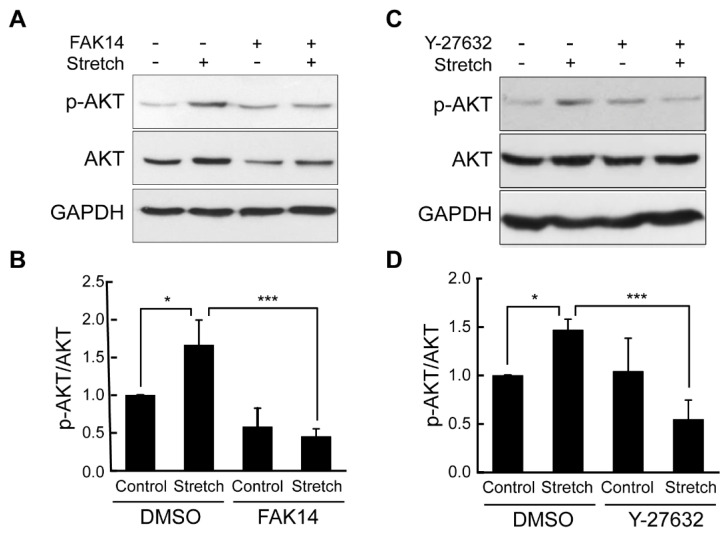
**Inhibiting FAK and ROCK abrogates stretch-induced AKT phosphorylation in adipocytes.** Adipocytes were stretched without or with FAK inhibitor (FAK14) (**A**,**B**) and ROCK inhibitor (Y-27632) (**C**,**D**). Immunoblots of AKT and p-AKT with GAPDH loading control. Relative band intensities were calculated the same way as in Figure 1. Mean ± SEM, *: *p* < 0.05 and ***: *p* < 0.001. n = 3 in (**B**) and n = 4 in (**D**). ANOVA/Fisher LSD post hoc test.

Finally, to assess the mediatory role of FAK and ROCK in the stretch regulation of AKT activation, mechanical stretch tests were conducted under FAK and ROCK inhibitors. Interestingly, cyclic stretch loading-induced AKT phosphorylation in adipocytes was significantly suppressed when adipocytes were stretched in the presence of FAK and ROCK inhibitors (Figure 4). Combined results suggest that FAK and ROCK in association with actin cytoskeletal architecture may function as mechanosensors for adipocytes under cyclic stretch loading to alter AKT insulin signaling.

## 4. Discussion

We applied physiologically relevant mechanical stretch loading to differentiated 3T3-L1 adipocytes to evaluate the stretch effect on basal AKT signaling and GLUT4 translocation. Cyclic stretching (5%, 1 Hz, 2 h) triggered AKT phosphorylation at Ser473 in adipocytes, a key insulin signaling effector. Further, GLUT4 localization in the plasma membrane, a downstream effector of AKT required for glucose uptake, was induced in cyclically stretched adipocytes. The investigation into the mechanosensing mechanism showed that inhibition of FAK, a tyrosine kinase that modulates focal adhesion/cytoskeleton signaling, impaired cyclic stretch-induced AKT phosphorylation. Similarly, inhibition of ROCK, a direct upstream effector of actin filament formation, eliminated stretch upregulation of AKT activation. This is the first study to report the role of FAK and ROCK mechanosensors in cyclic stretch control of adipocyte AKT phosphorylation. Our results suggest a new insight into unknown mechanical loading control of adipocyte insulin signaling. This may open a new horizon for adipocytic insulin metabolic mechanotransduction, which is in sharp contrast to the existing studies that have only explored the stretch loading control of adipogenic commitment and differentiation [6,7,8].

Insulin signaling plays a key role in glucose homeostasis. Insulin binds to the IR to initiate multiple phosphorylation cascades including insulin receptor substrate-1 (IRS-1), a predominant substrate found in adipose and muscle tissues. Once phosphorylated, IRS-1 activates phosphatidylinositol-3-kinase (PI3K), leading to AKT phosphorylation at Thr308 and Ser473, which is required for the translocation of glucose transporters to the plasma membrane [15]. Our result on the upregulated AKT phosphorylation at Ser473 by cyclic stretching (Figure 1) provides key evidence to suggest a role dynamic mechanical loading may play in glucose metabolism in adipocytes. While our finding is the first of its kind for adipocytes (cells not traditionally highlighted as mechanoresponsive), it aligns with stretch-induced AKT activation in muscle cells [16,17,18], another major cell type responsible for glucose metabolism. In addition to glucose homeostasis, stretch-induced AKT activation can regulate MSC anti-adipogenic differentiation [19], inhibit endothelial cell apoptosis [20], and mediate lung epithelial–mesenchymal transition [21], implying a potential universal role of mechanosensitive AKT activation.

Glucose uptake is mediated by glucose transporters, GLUT1 and GLUT4. GLUT4 is mobilized in response to insulin for glucose uptake in adipose, muscle, and liver tissues. The failure of GLUT4 to properly translocate from the perinuclear region to the plasma membrane results in impaired glucose metabolism and insulin resistance. In our study, GLUT4 expression in unstretched adipocytes was mostly localized around the nucleus and in the cytoplasm, while stretched adipocytes displayed increased GLUT4 expression at the plasma membrane (Figure 2A–C). It was reported that diminished glucose uptake in T2D could be due to reduced GLUT4 translocation rather than depleted GLUT4 levels [14]. Furthermore, our biotinylation test quantitatively evidenced that cyclic stretch significantly upregulated membrane surface expression of GLUT4, while its level in total lysates remained relatively unchanged (Figure 2D,E). Taken together, our data suggest that stretch might contribute to enhance glucose uptake in adipocytes via AKT-mediated increase in GLUT4 translocation to the plasma membrane but not by increasing total GLUT4 levels.

The mechanism by which mechanical stretch loading induces the AKT activation and its downstream effectors, including GLUT4, may lie with the action cytoskeleton. It has been established that, without mechanical loading, the actin network is required for insulin-mediated glucose metabolism [22]. In fact, for 3T3-L1 adipocytes, intact actin-network-facilitated subcellular transportation of PI3K to GLUT4 vesicles by insulin [23] and depolymerization of actin filaments impaired insulin-dependent AKT activation [24]. Similarly, for chondrocytes, an actin inhibitor (cytochalasin D) suppressed the phosphorylation of AKT and p38 mitogen-activated protein kinase (MAPK), key players in chondrocyte redifferentiation [25]. Our study further evidenced this role of actin to control AKT in adipocytes under mechanical stretch loading and identified potential regulatory mechanosensors such as FAK and ROCK (Figure 3 and Figure 4).

One crucial component by which the actin network in cells is altered is via integrin-mediated focal adhesion. Transmembrane integrins bind to ECM proteins to form focal adhesion sites, and focal adhesion proteins (talin, paxillin, vinculin, FAK, etc.) link integrins to the actin cytoskeleton to facilitate both outside-in and inside-out force transduction. FAK behaves as a central mediator of focal adhesion signaling to regulate various cellular processes, including, but not limited to, growth, differentiation, and motility [26]. While the control of insulin signaling via FAK has been proposed, the impact of this regulation may differ across cell types. A reduction in FAK in skeletal muscle cells led to impaired PI3K/AKT signaling and decreased insulin-mediated glucose transport, as associated with reduced actin filament formation [27]. Similarly, in FAK-silenced mice, AKT phosphorylation was significantly diminished in muscle and liver, leading to hyperglycemia and insulin resistance [28]. In contrast, for neurons, FAK was found to be a negative regulator of insulin-induced PI3K/AKT signaling [29]. While research on FAK in adipocytes is limited, one recent study showed that adipose-specific deletion of FAK causes increased inflammation, macrophage infiltration, and apoptosis [30]. Another study reported that adipose-selective inhibition of FAK activity through deleting β1 integrin results in a lipodystrophy-like character and insulin resistance in mice [31]. Regardless of these findings, almost nothing is revealed about the role of FAK in adipocyte insulin signaling under mechanical loading. Our data provide the first evidence that inhibition of FAK using the FAK14 inhibitor notably disrupts the actin filament network (Figure 3) and negates the positive effect of cyclic stretching on AKT phosphorylation in adipocytes (Figure 4A,B).

Another direct component that affects the actin network is RhoA/ROCK signaling. RhoA/ROCK induces actin filament formation and myosin-based contractility via LIMK and Cofilin and facilitates integrin-mediated focal adhesion, regulating a wide range of cellular functions, including adhesion, migration, and differentiation [32]. We recently showed that ROCK controls the internal cellular stiffness of adipocytes and its outward exertion of traction force [10]. This may suggest the role of Rho/ROCK-mediated force transmission in adipocyte metabolic mechanotransduction. Interestingly, the data published so far are rather contradictory. In 3T3-L1 adipocytes and L6 myotubes, inhibition of ROCK significantly impaired insulin-stimulated GLUT4 membrane translocation and glucose uptake [33], indicating a positive role of Rho/ROCK. On the other hand, RhoA-mediated glucose transport in adipocytes and myoblasts may occur independent of PI3K/AKT signaling [34]. Such a complexity in Rho/ROCK in insulin signaling observed under static culture conditions continues in studies utilizing mechanical loading. For instance, cyclic stretching-induced Rho/ROCK activation was positively associated with PI3K/AKT activation in rat aortic smooth muscle cells [35]. In contrast, cyclic stretching of human pluripotent stem cells activated Rho/ROCK while suppressing AKT phosphorylation [36]. Our study is the first to report the role of cyclic stretch in increasing AKT phosphorylation in adipocytes and to disclose the regulatory role of ROCK (Figure 4C,D) and the associated actin structure (Figure 3) in mediating this response. Overall, our study supports the positive impact of cyclic mechanical stretch loading on ROCK signaling and AKT activation for adipocytes.

## 5. Conclusions

Our study provides the first evidence of a stimulatory impact of cyclic mechanical stretch loading on AKT phosphorylation and GLUT4 plasma membrane translocation in differentiated 3T3-L1 adipocytes. FAK and ROCK inhibitors not only disrupted the actin network but also impaired stretch-induced AKT phosphorylation, implicating the potential role of stretch loading and associated focal adhesion/cytoskeleton signaling in promoting insulin-mediated glucose metabolism in adipocytes. Based on these proof-of-concept data, further research can be carried out to provide in-depth perspectives on how adipocytes adapt to extracellular mechanical loading cues and respond in insulin metabolism. Future studies will include experiments on varying stretch regimens while maintaining physiological relevancy, up- and downstream insulin signaling and metabolic effectors, basal and insulin-stimulated conditions, and potential coordination between FAK and ROCK. These findings may lay the groundwork for future in vivo studies to explore the therapeutic potential of mechanical loading in improving insulin sensitivity and glucose metabolism for obesity and T2D. For example, cyclic mechanical stretch loading reflective of dynamic exercise may provide a tool to improve insulin sensitivity in patients with obesity and T2D. Moreover, FAK and ROCK could be explored as regulatory molecular mechanosensing targets to invigorate the mechanical loading-induced insulin signaling activation. Ultimately, adjusting adipocytic metabolic adaptation capacity to physiological mechanical loading milieus via manipulating associated mechanosensors may suggest a new insight and approach to address metabolic disorders.

## Data Availability

The data that support the findings of this research are available upon reasonable request to the corresponding author.

## Supplementary Materials

The following supporting information can be downloaded at: https://www.mdpi.com/article/10.3390/bioengineering11121279/s1, Figure S1. Adipogenic differentiation of 3T3-L1 preadipocytes. (A) Oil red O staining for assaying lipid accumulation. (B) Stained oil red O was extracted with isopropanol and absorbance was measured using a spectrophotometer (BioTek) at an absorbance of 490 nm. (C) Immunoblots of FAS and C/EBPα for adipogenic protein expression. (D,E) Relative immunoblot band intensities were compared after normalization with GAPDH. Mean ± SEM, *: *p* < 0.05 and **: *p* < 0.01 (n = 3) by Student *t*-test; Figure S2. ImageJ analysis of GLUT4 immunofluorescence images. Fixed adipocytes were incubated with a polyclonal anti-GLUT4 antibody followed by Alexa488-conjugated secondary antibody (green). Images were imported into ImageJ, and a line was drawn radially from inside the cell and across the cell membrane (shown in yellow). Only sections of the plasma membrane that were not directly adjacent to another cell were analyzed. Using the Plot Profile tool, GLUT4 intensity was plotted against distance (see the plot inset). The plot values were imported into Excel, converted into μm, and re-plotted.
