# Supplementary material for: Mechanical Stretch Control of Adipocyte AKT Signaling and the Role of FAK and ROCK Mechanosensors
Source: Bioengineering (Basel). 2024 Dec 16;11(12):1279. doi: 10.3390/bioengineering11121279 (PMC11673816; doi:10.3390/bioengineering11121279)
Supplement: Supplementary file 1 [file bioengineering-11-01279-s001.zip › bioengineering-3316041-supplementary.pdf]

Supplementary Data

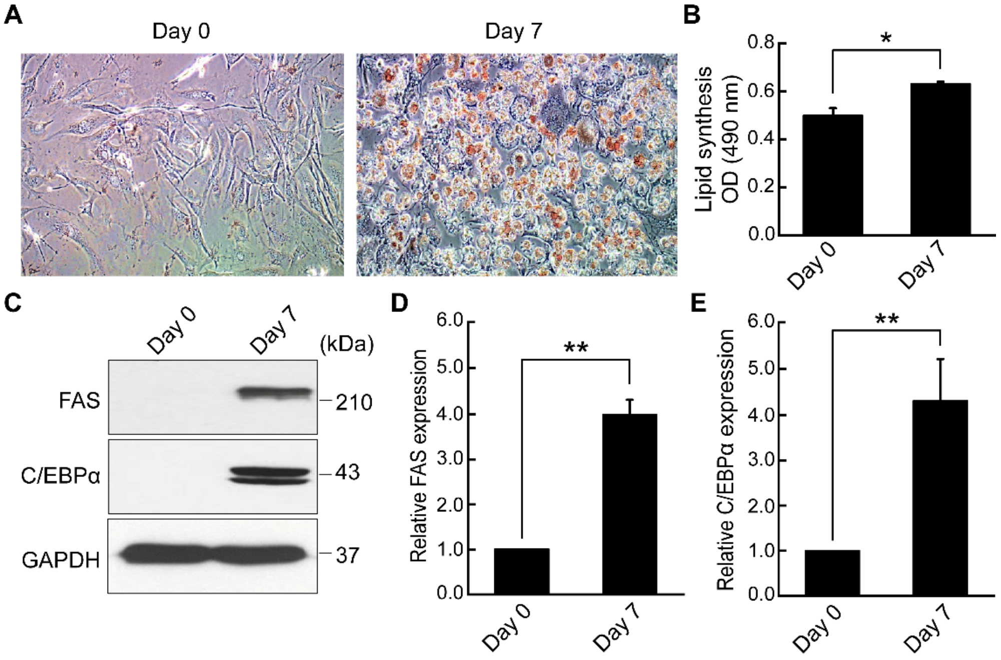

Figure S1. Adipogenic differentiation of 3T3-L1 preadipocytes. (A) Oil red O staining for assaying lipid accumulation. **(B)** Stained oil red O was extracted with isopropanol and absorbance was measured using a spectrophotometer (BioTek) at an absorbance of 490 nm. **(C)** Immunoblots of FAS and C/EBPα for adipogenic protein expression. **(D,E)** Relative immunoblot band intensities were compared after normalization with GAPDH. Mean ± SEM, *: $p < 0.05$ and **: $p < 0.01$ ($n = 3$) by Student t-test.

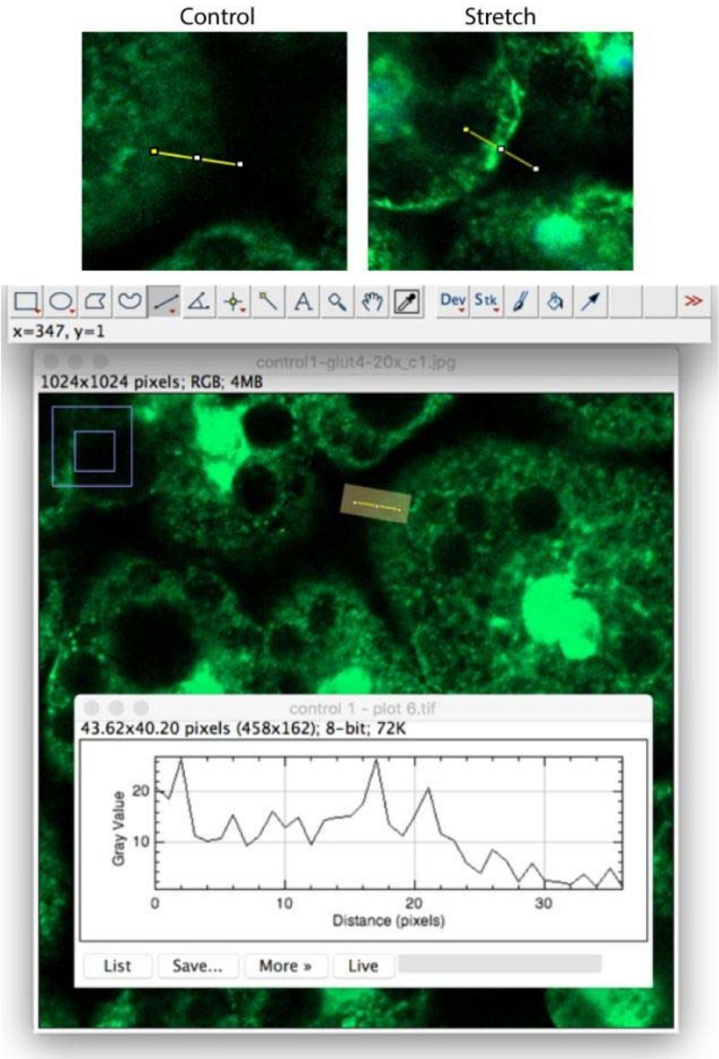

Figure S2. ImageJ analysis of GLUT4 immunofluorescence images. Fixed adipocytes were incubated with a polyclonal anti-GLUT4 antibody followed by Alexa488-conjugated secondary antibody (green). Images were imported into ImageJ, and a line was drawn radially from inside the cell and across the cell membrane (shown in yellow). Only sections of the plasma membrane that were not directly adjacent to another cell were analyzed. Using the Plot Profile tool, GLUT4 intensity was plotted against distance (see the plot inset). The plot values were imported into excel, converted into µm, and re-plotted.
